# Role of Oxidized Lipids in Permeation of H_2_O_2_ Through a Lipid Membrane: Molecular Mechanism of an Inhibitor to Promoter Switch

**DOI:** 10.1038/s41598-019-48954-z

**Published:** 2019-08-29

**Authors:** Yuya Ouchi, Kei Unoura, Hideki Nabika

**Affiliations:** Department of Material and Biological Chemistry, Faculty of Science, Yamagata University, 1-4-12 Kojirakawa, Yamagata, 990–8560 Japan

**Keywords:** Membrane biophysics, Membrane structure and assembly

## Abstract

H_2_O_2_ permeation through a cell membrane significantly affects living organisms, and permeation is controlled by the physico-chemical nature of lipids and other membrane components. We investigated the molecular relationship between H_2_O_2_ permeation and lipid membrane structure using three oxidized lipids. POVPC and PazePC act as intra- and inter-molecular permeation promoters, respectively; however, their underlying mechanisms were different. The former changed the partition equilibrium, while the latter changed the permeation pathway. PoxnoPC inhibited permeation under our experimental conditions via an intra-molecular configuration change. Thus, both intra- and inter-molecular processes were found to control the role of oxidized lipids as inhibitors and promoters towards H_2_O_2_ permeation with different mechanisms depending on structure and composition. Here, we identified two independent H_2_O_2_ permeation routes: (i) permeation through lipid membrane with increased partition coefficient by intra-molecular configurational change and (ii) diffusion through pores (water channels) formed by inter-molecular configurational change of oxidized lipids. We provide new insight into how biological cells control permeation of molecules through intra- and inter-molecular configurational changes in the lipid membrane. Thus, by employing a rational design for both oxidized lipids and other components, the permeation behaviour of H_2_O_2_ and other ions and molecules through a lipid membrane could be controlled.

## Introduction

Cell membrane acts as a physical barrier that prevents the exchange of ions and molecules between a cell’s internal and external environments. However, it is also necessary for cells to incorporate required substances and discharge unnecessary ones. Such a molecular exchange system is achieved through either active or passive permeation^1,2^. The former needs energy to transport ions and molecules against their concentration gradients. On the other hand, passive permeation involves spontaneous transport without the expenditure of energy. The essential mechanism of passive permeation without energy requirement originates from the fact that mass transportation is driven by multistep equilibria outside the cell – outer leaflet – inner leaflet – inside the cell^3,4^. Molecules diffuse from outside the cell partitions into the outer leaflet of the cell membrane according to its partition equilibrium. Then, penetrated molecules diffuse from the outer to inner leaflet, and finally escape from the inner leaflet to inside the cell according to its partition equilibrium. Since, the partition coefficient is determined by a balance of chemical and physical characteristics of two phases, passive permeation is strongly sensitive to the chemical and physical nature of both permeating molecule and lipid bilayer. Thus, the permeation behaviours of various substances, such as water^5–7^, sodium ion^8,9^, dyes^10,11^, antibiotics^12^, fatty acyl compounds^4^, and other small molecules^3,13–15^, have been investigated under various conditions of temperature (phase state)^8–10,16^, membrane composition^6,10,11^, membrane asymmetry^5^, and local heating by plasmonics^7^. For example, promotion of water permeation at phase transition temperature has been extensively investigated^8–10,16^, where gel/fluid interface with a higher partition coefficient has been proposed to play a dominant role.

Hydrogen peroxide (H_2_O_2_), a reactive oxygen species (ROS), is generated by mitochondrial respiration and has significant effects in living organisms, such as cytotoxic effect, apoptotic effect, oxidative stress, and vascular remodelling^17–19^. Since the diffusion of H_2_O_2_ through cell membrane is a key regulator in H_2_O_2_ affecting these biological processes, membrane transport of H_2_O_2_ has been investigated^20^. From both experiments and numerical simulations, the activation energy for H_2_O_2_ permeation has been estimated as 30–40 kJ/mol^21,22^, which is comparable to the activation energy of water permeation^13,23,24^, but is higher than those of other ROS^21,25,26^ and other molecules^3,4,13^. Higher activation energy of H_2_O_2_ permeation is due to the hydrophilic nature of the H_2_O_2_ molecule. Since, the inside of the cell membrane is filled with hydrophobic alkyl chains, hydrophilic H_2_O_2_ molecules hardly partition into the hydrophobic membrane inside. In other words, introduction of a hydrophilic moiety inside the hydrophobic alkyl chain region increases the partition coefficient and thus promotes the passive permeation of H_2_O_2_ molecules.

One important origin of hydrophilicity in the alkyl chain region is the incorporation of an oxidized lipid that bears a hydrophilic group at its alkyl chains. Various papers have reported an effect of oxidized lipids on the structure^27–34^, fluidity and diffusivity^35,36^, phase state^37^, and flip-flop dynamics^38^ of a cell membrane. Concerning passive permeation through cell membranes, an influence of the oxidized lipid has also been investigated by both experiments and simulations^29,30,32,39–41^, especially focusing on its effect on intra-molecular and an inter-molecular structural changes. The former involves the reorientation of the oxidized tails (*sn*-2 acyl chains) toward the water/membrane interface to form hydrogen bonds with water and the polar headgroup of surrounding lipids^27,28,32,33^. The reorientation angle between the vector from the first carbon to the oxidized carbon and the normal bilayer has been calculated to be in the range of 96–118 ° for both shorter and longer oxidized carbon chains, indicating that most oxidized tails prefer to undergo intra-molecular reorientation and form a looped structure by bending their oxidized moiety toward the water/membrane interface^27^. The appearance of intra-molecular re-orientation has been confirmed by the change in average molecular area^27,32,33^. On the other hand, more drastic change can be induced by the inter-molecular configurational change in the membrane. Similar to a pore-forming peptide such as magainin, the oxidized lipids are known to form nanoscopic pores that permit small molecules to pass between the inside and outside of the cell^29–32,34,42^. The appearance of a pore in the intact membrane has been confirmed by lipid oxidation by oxygen radicals, where nanopores with the diameter of 10–50 nm formed in a phospholipid bilayer^31^. Pore formation by the incorporation of oxidized lipids has also been experimentally observed on a giant unilamellar vesicle^42^. Since the ability to form inter-molecular hydrogen bonds is a critical parameter to determine the pore-forming ability, a strong influence on the nature of oxidized lipids or membrane composition have been reported^30,34^. A molecular dynamics study has revealed that pore formation proceeds based on the inter-molecular process following the intra-molecular process that is described above^32^. After the oxidized lipid forms the looped structure by bending its alkyl tails to the water/membrane interface (intra-molecular process), the oxygen of the oxidized molecule undergoes self-assembly to form a pore by forming hydrogen bonds with water and the polar headgroup of surrounding lipids (inter-molecular process). Thus, pore formation can be regarded as a two-step process. However, it is hard to clarify the dominant process that acts as the permeation inhibitor and promoter from experimental viewpoints. Furthermore, the permeation behaviour of H_2_O_2_ associated with intra- and inter-molecular configurational change of the oxidized lipids is still unclear. Therefore, the present study investigated the relationship between the permeation of H_2_O_2_ and the structure of lipid membranes, by using three different oxidized lipids 1-palmitoyl-2-(5′-oxo-valeroyl)-sn-glycero-3-phosphocholine (POVPC), 1-palmitoyl-2-(9′-oxo-valeroyl)-sn-glycero-3-phosphocholine (PoxnoPC), and 1-palmitoyl-2-azelaoyl-sn-glycero-3-phosphocholine (PazePC). The permeation of H_2_O_2_ through 1,2-dioleoyl-sn-glycero-3-phosphocholine (DOPC) liposomes doped with one of these oxidized lipids was characterized with a spectrofluorometer using a liposome that emits chemiluminescence in the presence of H_2_O_2_. Change in the intra- and inter- molecular configuration was traced by a pressure-area (π - A) isotherm, which enables the characterization of the structural change at a molecular scale. By correlating the permeation and the molecular area occupied by the oxidized lipids, it became possible to clarify the effects of intra- and inter-molecular changes of the oxidized lipids on their role as permeation inhibitor and promoter. By comparing three oxidized lipids with different chain lengths and oxidized groups, the molecular mechanism underlying permeation inhibition and promotion through lipid membrane was discussed by correlating the change in the permeation behaviour induced by intra- or inter-molecular changes of the oxidized lipids. Although the present study focused on permeation of the H_2_O_2_ molecule, our finding on the relation between permeation and structure can be applied to other ions and molecules as well.

## Materials and Methods

**Figure 1:**
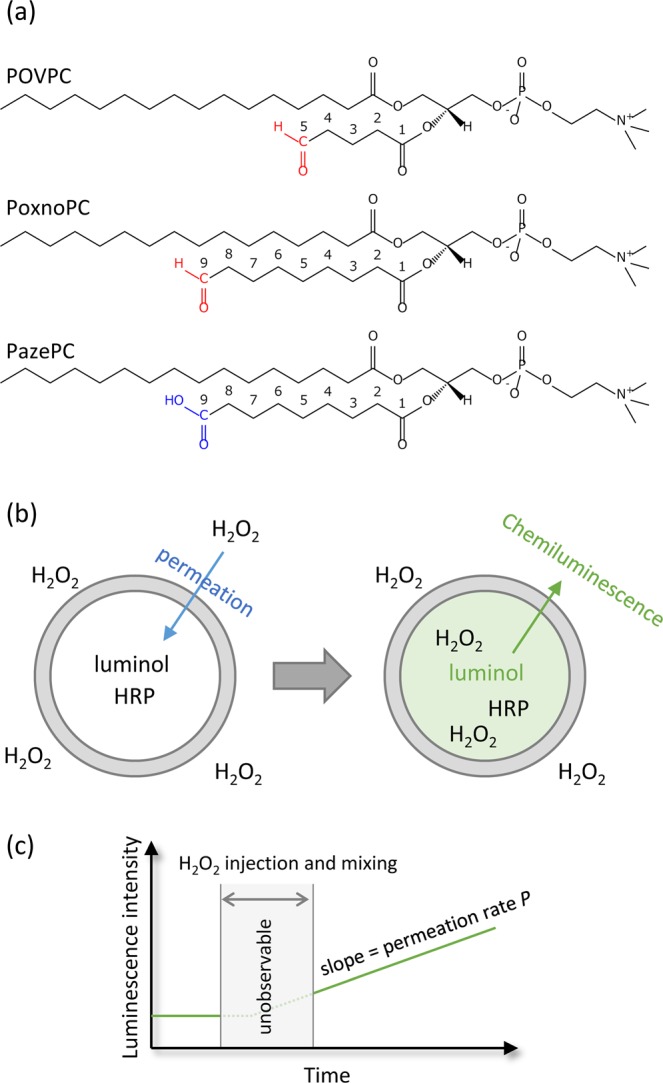
(**a**) Structures of oxidized lipids used in the present study. (**b**) Detection of H_2_O_2_ permeation by chemiluminescence in a liposome incorporated with luminol and HRP. (**c**) Measurement of chemiluminescence by spectrofluorometer, where the slope in the chemiluminescence intensity is defined as the permeation rate *P* and used as a measure of the permeability of H_2_O_2_.

All chemicals and solvents were commercially available and used as received without any treatment. DOPC, POVPC, PoxnoPC, and PazePC were purchased from Avanti Polar Lipids Ltd. (Alabaster, AL, U.S.A.). Structures of three oxidized lipids used in the present study is shown in Fig. 1a. Luminol and horseradish peroxidase (HRP) were purchased from Nacalai Tesque, Inc. (Kyoto, Japan) and Wako Pure Chemical Industry (Tokyo, Japan), respectively.

Chloroform solutions of DOPC (10 mg/mL) and one of the oxidized lipids (1 mg/mL) were mixed in a glass vial, and the solvents were evaporated by a nitrogen gas blow followed by evacuation in a vacuum desiccator for over 7 h. We prepared a chemiluminescent liposome suspension by agitating the vacuum-dried lipid films in a mixture of 2.0 mL of buffer solution (50 mM Tris/HCl, 10 mM luminol, pH 8.6) and 0.5 mL of HRP solution (100 μM). The solution was then treated by vortexing at 45 °C, freeze-thawing, and extruding through a 100 nm and 80 nm polycarbonate filters. Chemiluminescent liposomes incorporating luminol and HRP were separated from free luminol and HRP by density gradient centrifugation (DGC). A density gradient column was formed by layering, from bottom to top, 1.2 mL of 60% sucrose solution containing liposome, 1.5 mL of 30% sucrose solution, 1.5 mL of 10% sucrose, and 1.0 mL of 50 mM Tris/HCl (pH 8.6) solution. DGC was performed at 604,000 *g* for 1 h. After centrifugation, the layer with liposome was extracted and diluted by the buffer solution.

Permeation of H_2_O_2_ through a lipid membrane was analysed by chemiluminescence that is emitted from luminol and HRP present inside the liposome (Fig. 1b). The chemiluminescence was measured with a spectrofluorometer FP-6300 (JASCO, Tokyo, Japan) by adding 50 μL of 8.8 mM H_2_O_2_ solution into 100 μL of dispersion solution of chemiluminescent liposome. The exciting wavelength was 425 nm and the band width was 20 nm. Although the liposome emitted chemiluminescence just after the injection and mixing of H_2_O_2_ into a liposome dispersion solution, the data was not acquired during these operations because the spectrometer’s lid was open. After closing the lid, a continuous increase in chemiluminescence was observed by a continuous permeation and accumulation of H_2_O_2_ inside the liposome. The slope in the chemiluminescence intensity was defined as the permeation rate *P* and used as a measure of the permeability of H_2_O_2_ for further analysis (Fig. 1c). Representative experimental results showing the continuous increase in chemiluminescence intensity are shown in Figure S1 in the Supplementary Information.

π–A isotherms were measured at the air-water interface with Minitrough System 3 (KSV Ltd., Helsinki, Finland) equipped with two moving barriers and a Pt Wilhelmy plate. After filling the trough with the buffer solution (200 mL, 50 mM Tris/HCl (pH 8.6)), the solution was settled by heating to 25 °C using a circulating water bath system. The buffer solution (30 mL) was removed by an aspirator to remove contaminations at the air/water interface. Then, a chloroform solution of the lipid was spread on the subphase using a Hamilton microsyringe. After waiting for 10 min for the solvent to evaporate, the lipid was compressed at a constant rate of 5 mm/min.

## Results and Discussion

**Figure 2:**
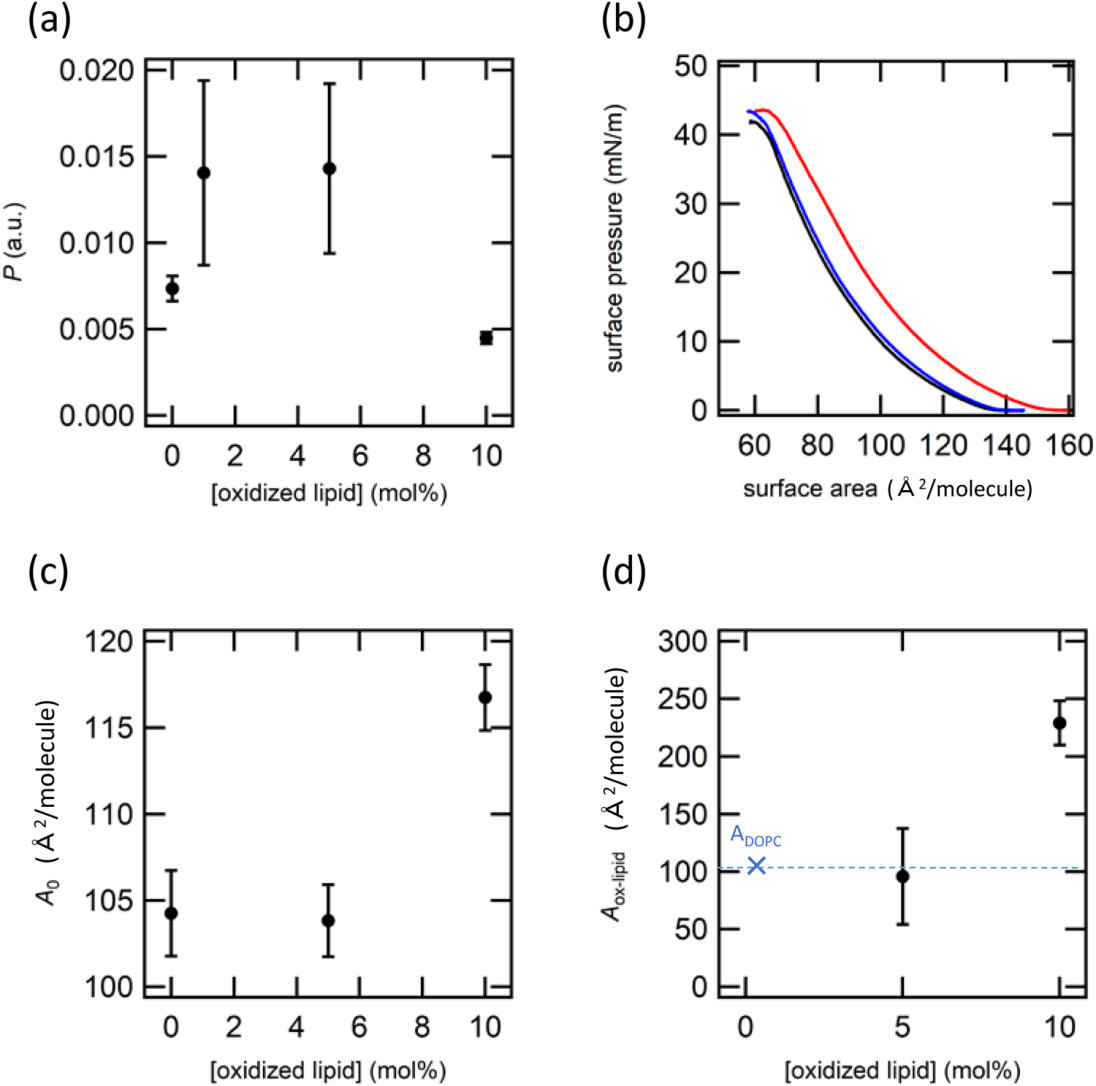
Data for POVPC. The permeation rate *P* as the function of the concentration of POVPC. (**b**) *π* – *A* curves of DOPC/POVPC monolayers. The concentrations of 5CHO-PC are 0 (black), 5 (blue), and 10 (red) mol %. (**c**) Averaged limiting area (*A*_0_) estimated from π – A curves as the function of the concentration of POVPC. (**d**) Calculated limiting area of oxidized lipid (*A*_ox-lipid_) as the function of the concentration of POVPC.

Figure 2 shows experimental data for DOPC membrane doped with POVPC at various concentrations. H_2_O_2_ permeation rate *P* first increases from 0.007 to 0.015 with the addition of POVPC until its molar concentration exceeds 5 mol %, which indicates that the presence of POVPC below 5 mol % enhances passive permeation of H_2_O_2_ molecules (Fig. 2a). As mentioned above, changes in passive permeation can be discussed in terms of the change in the partition equilibria between the aqueous phase and lipid membrane. Promotion of H_2_O_2_ permeation is explained by an increased partition of H_2_O_2_ molecule from the outside of the liposome to the membrane, where the H_2_O_2_ molecules in the membrane finally dissolved into the interior of the liposome. As a result, increased permeation of H_2_O_2_ was detected as increased chemiluminescence inside the liposomes. However, further increase in POVPC to 10 mol % decreased *P* to 0.005, which was lower than the level of POVPC -free DOPC. Transition from increased to decreased permeation implies the presence of two competitive roles of POVPC as the permeation promoter and inhibitor, which switched depending on the molecular composition.

To clarify the mechanism of promoter/inhibitor transition, we characterized the membrane structure at a molecular scale (Fig. 2b). π-A measurement of DOPC exhibits typical curve of a liquid expanded phase without obvious phase transition to a liquid crystal phase. Addition of POVPC at 5 mol % did not change the π-A curve, whereas 10 mol % of POVPC shifted the π-A curve to higher area side. From the π-A measurements, a limiting area (*A*_0_) of each condition was estimated (Fig. 2c). As expected from the π-A curves, *A*_0_ did not change below 5 mol % but increased at 10 mol %. It should be noted that the value of *A*_0_ is a number-averaged area occupied by DOPC and POVPC in the bilayer. Thus, we quantitatively estimated an occupied area by the oxidized lipid (*A*_ox-lipid_) with the following equation, by assuming that the occupied area by DOPC (*A*_DOPC_) was insensitive to the presence of oxidized lipids.

1 $${A}_{0}=(1-x){A}_{DOPC}+x{A}_{ox-lipid},$$

where *x* is the molar fraction of oxidized lipid in the membrane. *A*_ox-lipid_ at 5 mol % was almost the same as *A*_DOPC_, indicating that POVPC distributed in the DOPC bilayer with the same structural configuration as DOPC that has one head group exposed to the aqueous phase and two alkyl chains separated from the aqueous phase (Fig. 2d). On the other hand, *A*_ox-lipid_ increased more than twice at 10 mol % from 104 Å^2^ to 229 Å^2^. Such a large change in *A*_ox-lipid_ can be explained by a change in the molecular configuration of POVPC in the DOPC bilayer. Previous reports based on molecular dynamic simulations proved that hydrophilic aldehyde group tends to escape from a hydrophobic alkyl region to a hydrophilic head group region by the intra-molecular configuration change^27,28,32,33^. Since such looped structure increases the molecular area occupied by the oxidized lipid, the increased *A*_ox-lipid_ at 10 mol % can be interpreted in terms of the formation of the looped structure. Although the reason why the looped structure did not appear until the concentration of POVPC increased to 10 mol % is still unclear, this concentration dependence would be related to a kind of phase transition that changes molecular distribution, configuration, hydrogen bond network, or mobility with sharp changes in concentration. From these experiments, it was found that POVPC in DOPC acts as a permeation promoter when POVPC has a non-looped structure similar to DOPC, whereas its role switches from promoter to inhibitor by changing molecular configuration from non-looped to looped structure at higher molecular concentration.

**Figure 3:**
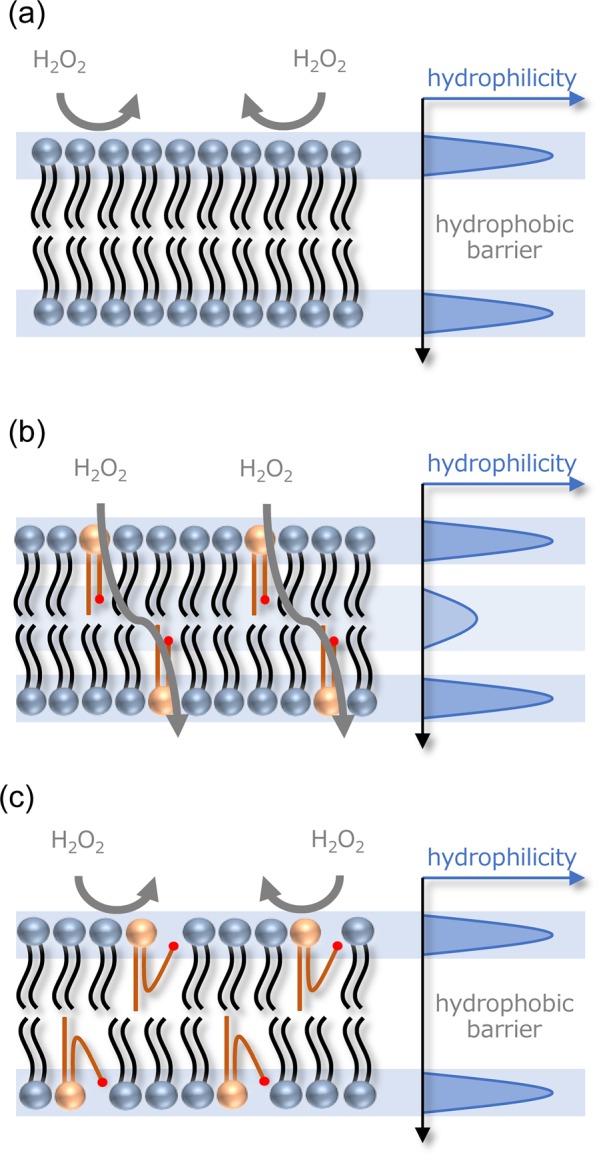
Schematic illustration of H_2_O_2_ permeation through DOPC/POVPC bilayer under different molecular configurations. (**a**) DOPC bilayer without POVPC has a high hydrophobic barrier at the alkyl chain region, which inhibits the permeation of H_2_O_2_. (**b**) POVPC at 5 mol % forms a molecular configuration similar to DOPC that has nonlooped alkyl chains, which promotes the permeation of H_2_O_2_ through the bilayer. (**c**) POVPC at 10 mol % forms a looped structure, which forms again high hydrophobic barrier to reduce the permeation of H_2_O_2_.

Relationship between the role (promoter or inhibitor) and structure (non-looped or looped structures) is schematically illustrated in Fig. 3. It is well known that a lipid bilayer acts as a physical barrier that prevents permeation of molecules across the bilayer, especially for hydrophilic molecules because of the presence of a hydrophobic barrier inside the bilayer (Fig. 3a). When oxidized lipid is incorporated and adapts the same configuration as the other lipids, the hydrophilic groups such as aldehydes are located at the hydrophobic region. This configuration reduces the hydrophobic barrier. Under this configuration, the oxidized lipids act as a promoter for hydrophilic molecules such as H_2_O_2_ to penetrate into the bilayer. Partitioned H_2_O_2_ at the alkyl region finally diffuses into the inside of liposome according to its partition equilibrium between the membrane and inside the liposome. Thus, the incorporation of the hydrophilic region inside the bilayer shifts the distribution of H_2_O_2_ from the outside to inside of the liposome, which was observed as enhanced permeation at 5 mol %. However, the looped structure at 10 mol % cannot bring hydrophilicity inside the bilayer. The hydrophobic barrier persists similar to neat DOPC bilayer without oxidized lipids. Thus, the bilayer is still hydrophobic and does not promote the permeation of H_2_O_2_. Furthermore, suppressed permeation compared to the neat DOPC bilayer (Fig. 2a) would be related to the presence of the aldehyde group at the water/bilayer interface. Phosphatidylcholine has been reported to play a role in the decomposition of H_2_O_2_, which was found to be triggered through interactions between H_2_O_2_ and polar head group of phosphatidylcholines^43^. Especially, the packing density of phosphatidylcholines critically influenced the reactivity, which was confirmed by its temperature dependence and assembly dependence (vesicles *vs*. micelles *vs*. monomers). Clearly, decomposition of H_2_O_2_ at the polar head group region decreases the net permeability of H_2_O_2_. Presence of more reactive states at the water/membrane interface further decreases the net permeability. Thus, suppressed permeability at 10 mol % compared to neat DOPC bilayer can be explained by the formation of a more reactive state by the intra-molecular reorientation of the oxidized tails toward the water/membrane interface to form hydrogen bonds with water and the polar headgroup of surrounding lipids. This can be further understood by structural analyses using infrared (IR) and other spectroscopies in future.

**Figure 4:**
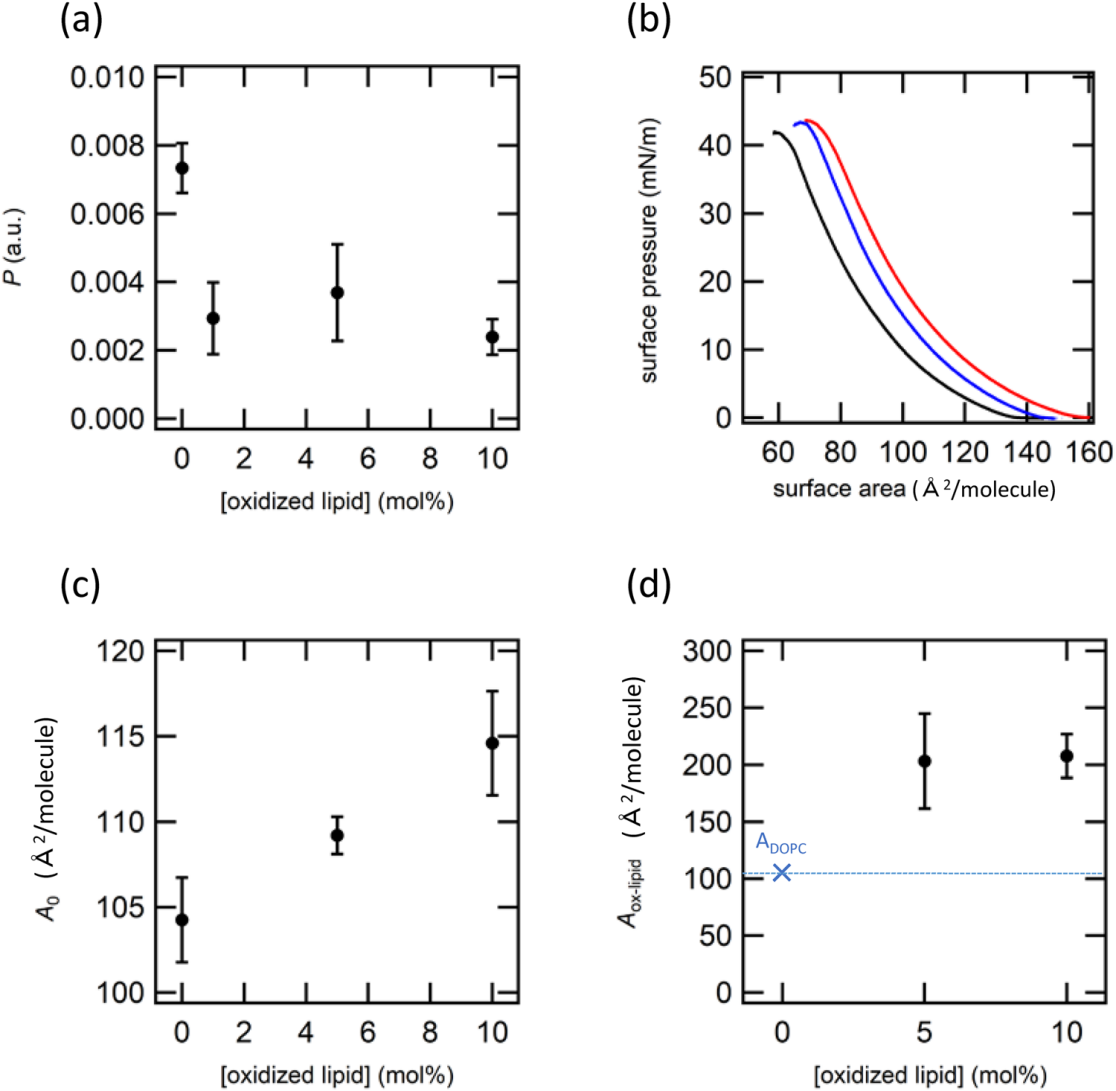
Data for PoxnoPC. The permeation rate *P* as the function of the concentration of PoxnoPC. (**b**) *π* – *A* curves of DOPC/PoxnoPC monolayers. The concentrations of PoxnoPC are 0 (black), 5 (blue), and 10 (red) mol %. (**c**) Averaged limiting area (*A*_0_) estimated from π – A curves as the function of the concentration of PoxnoPC. (**d**) Calculated limiting area of oxidized lipid (*A*_ox-lipid_) as the function of the concentration of PoxnoPC.

Since it has been reported that oxidized lipids with different structure and length of the oxidized alkyl chain exerted significantly different effects on the physicochemical properties of the lipid membrane^44,45^, we have compared the effect of oxidized lipids with different structures. Figure 4 shows the experimental data for DOPC bilayer doped with PoxnoPC that has longer alkyl chain compared with POVPC, but with an aldehyde group at its end similar to POVPC. Contrary to the permeation activity of POVPC that switched between inhibitor and promoter roles depending on its molecular concentration, PoxnoPC showed only inhibitory activity in the range of up to 10 mol % (Fig. 4a), where the permeation rate *P* decreased from 0.007 to 0.003. Under these concentrations, π-A curves showed a gradual shift to the larger area side (Fig. 4b) and the averaged limiting area *A*_0_ demonstrated a consistent increase (Fig. 4c) with an increase in the concentration of PoxnoPC. *A*_ox-lipid_ of PoxnoPC exhibited a significant expansion to 205 Å^2^ at both 5 and 10 mol % that is comparable to *A*_ox-lipid_ of POVPC at 10 mol % (Fig. 4d). This result indicates that PoxnoPC undergoes intra-molecular configuration change and adopts the looped structure at lower concentration compared with POVPC, which is consistent with the fact that PoxnoPC exhibited only inhibitive effect at lower concentration. Promotion of the formation of looped structure of PoxnoPC can be explained by the more flexible nature of longer alkyl chain of PoxnoPC that reduces an energy barrier to bend the alkyl chain to form the looped structure. In analogy with the inhibitory activity of POVPC at 10 mol %, the role of PoxnoPC as the permeation inhibitor at any concentration in the present experiment would thus be attributed to the intra-molecular changes to form the looped structure.

**Figure 5:**
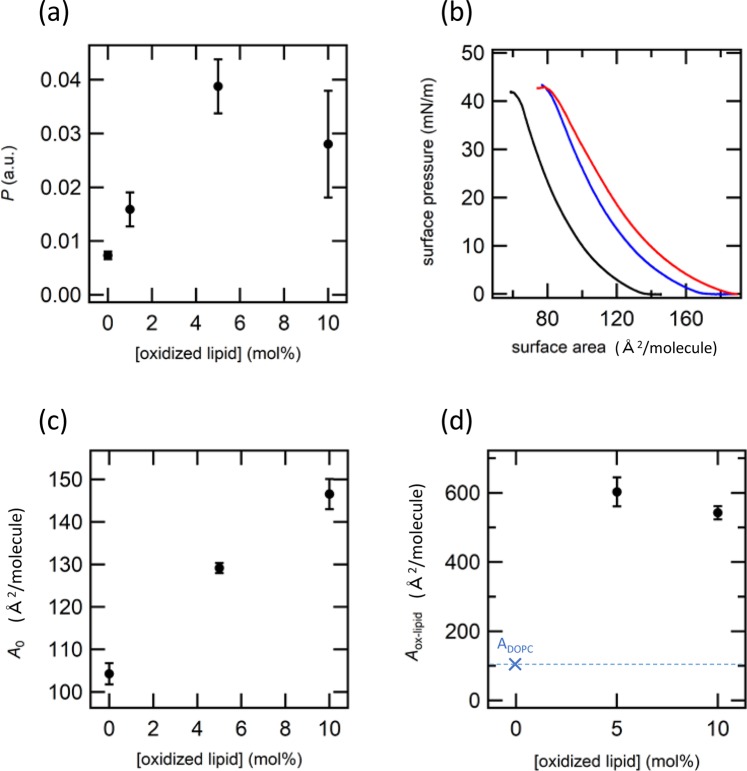
Data for PazePC. The permeation rate *P* as the function of the concentration of PazePC. (**b**) *π* – *A* curves of DOPC/PazePC monolayers. The concentrations of 9COOH-PC are 0 (black), 5 (blue), and 10 (red) mol %. (**c**) Averaged limiting area (*A*_0_) estimated from π – A curves as the function of the concentration of PazePC. (**d**) Calculated limiting area of oxidized lipid (*A*_ox-lipid_) as the function of the concentration of PazePC.

**Figure 6:**
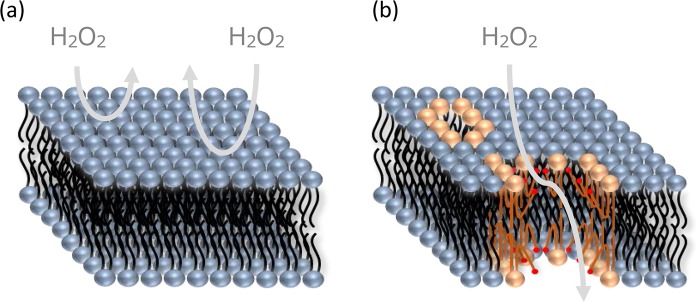
Schematic illustration of H_2_O_2_ permeation through DOPC/PazePC bilayer. (**a**) DOPC bilayer without PazePC has high hydrophobic barrier at the alkyl chain region, which inhibits the permeation of H_2_O_2_. (**b**) PazePC undergoes self-assembly to form a hydrophilic pore that can act as a channel for hydrophilic H_2_O_2_.

Finally, we investigated the effect of functional groups of the oxidized lipid without changing the chain length. Figure 5 shows the experimental data of DOPC doped with PazePC at different concentrations. The permeation rate *P* was increased by the addition of PazePC at any concentration, indicating that PazePC acted as the permeation promoter (Fig. 5a). Furthermore, *P* in the presence of PazePC (above 0.015) was significantly higher than other oxidized lipids investigated in the present study. To clarify the origin of such high promotive effect of PazePC, we have characterized the structure by π-A measurements (Fig. 5b). Similar to PoxnoPC (Fig. 4b), π-A curves gradually shifted toward larger area side. However, the shift was more significant compared to PoxnoPC. Quantitative analysis clearly showed a significant increase in the averaged limiting area *A*_0_ from 115 Å^2^ to 145 Å^2^ at 10 mol %, which was higher than 115–120 Å^2^ for POVPC and PoxnoPC, respectively. It should be noted that the increase by 20–30 Å^2^ was brought from the presence of as small as 10% of PazePC, implying that the area increase per single PazePC might be much larger. Actually, numerical analysis based on Eq (1) revealed that the occupied area by single PazePC (*A*_ox-lipid_) was over 500 Å^2^, which is five times larger than that of DOPC (*A*_DOPC_). This result indicates that the observed area expansion by PazePC cannot be accounted by the intra-molecular conformational change of a single PazePC molecule. Instead, formation of a self-assembled structure that forms a pore by the inter-molecular configurational change in the membrane would be a plausible mechanism that can account for both significant permeation and area expansion (Fig. 6). This behaviour is consistent to the previous result of pore formation that has been observed by atomic force microscopy^31^ and molecular dynamic simulations^29,30,34^. Our assumption that PazePC underwent inter-molecular change to form pores can be supported by the fact that PazePC demonstrated a significantly higher role as a permeation promoter, because the pores form a water channel that allow molecules to diffuse between the outside and inside of the liposome without any energy barrier. This process has kinetic superiority compared with permeation through a lipid membrane, which is the cause of high permeability in the presence of PazePC. Thus, in contrast to POVPC, that shows promoting activity by intra-molecular change, the origin of the enhanced permeability of PazePC would be attributed to the inter-molecular configurational change. From this finding, it was suggested that the origin of inhibition or promotion for the molecular permeation of lipid membrane should be discussed considering two independent molecular processes; intra- and inter-molecular processes.

**Figure 7:**
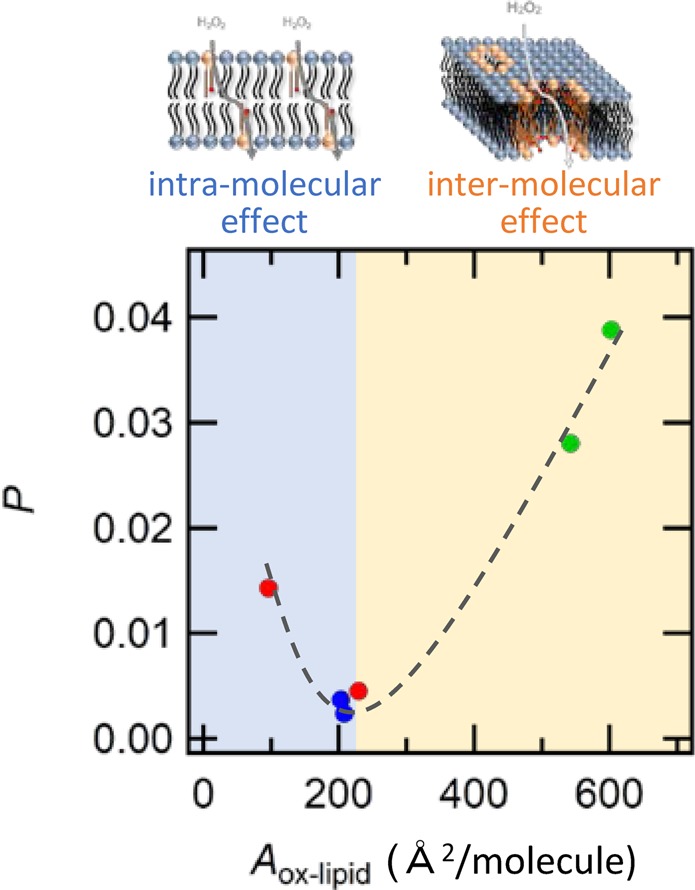
Relationship between the permeation rate *P* and limiting area of oxidized lipid *A*_ox-lipid_ of POVPC (red), PoxnoPC (blue), and PazePC (green).

Correlation between the permeation rate and membrane structure at molecular scale further clarified the permeation mechanisms that are associated with the intra- and inter-molecular processes (Fig. 7). Permeation minimum appears at *A*_ox-lipid_ = 200 Å^2^, whose *P* was almost the same as *P* of DOPC membrane without oxidized lipids. Under this condition, the oxidized lipids act as the permeation inhibitor by placing the hydrophilic oxidized group near the region of lipid head groups at the water/membrane interface. On the other hand, when the oxidized group could not adopt the looped structure and *A*_ox-lipid_ became smaller, the role as permeation inhibitor became weakened. Instead, incorporating the oxidized group inside the alkyl chain weakened the hydrophobic barrier, which increased the partition coefficient of H_2_O_2_ into the bilayer. Enhanced H_2_O_2_ partition resulted in the increase in *P* at smaller *A*_ox-lipid_ side. Switching between looped and nonlooped structure is an intra-molecular process, which can be controlled through hydrophilicity and alkyl chain length of the oxidized group. Higher numbers of hydrophilic oxidized groups with longer alkyl chain tends to form the looped structure that makes the oxidized lipids act as permeation inhibitor. On the other hand, the oxidized lipids with *A*_ox-lipid_ larger than 500 Å^2^ also demonstrated increased H_2_O_2_ permeation. This process cannot be explained by the intra-molecular process, instead, an inter-molecular process would be the dominant mechanism in this scenario. Previous papers have suggested that the pore formation proceeds by inter-molecular self-assembly following the intra-molecular process^29–32,34,42^. Assuming that each oxidized lipid adopts the looped structure via the intra-molecular process, net area occupied by the oxidized lipid that excludes the area of vacancy would be similar to the value of *A*_ox-lipid_ for the looped structure (ca 200 Å^2^). Thus, when *A*_ox-lipid_ is larger than 500 Å^2^, the remaining area larger than 300 Å^2^ corresponds to the vacancy occupied by each looped molecule formed in the membrane. The area of pore that is formed by the inter-molecular self-assembly associating *n* molecules can be roughly estimated as 300 × *n* Å^2^. A 10-molecular pore therefore gives 3,000 Å^2^, which is comparable to the area of pores during plasma oxidation^31^. Thus, it is plausible to assume that the observed large *A*_ox-lipid_ corresponds to the formation of pores that are formed by the inter-molecular self-assembly. Through pores of a few tens of nanometres, leakage of the luminol and HRP from chemiluminescent liposome into bulk aqueous phase should be also considered. The leakage of these substances reduces the chemiluminescence intensity from the liposome, as the leaked luminols would not contribute to the chemiluminescence intensity because they are too diluted in the bulk aqueous phase to react with H_2_O_2_ catalysed by HRP. Thus, the leakage totally reduces the net chemiluminescence intensity, which indicates that the permeation rate *P* under the pore-forming condition would be underestimated. However, it is still plausible to conclude that permeation under the inter-molecular pore-forming conditions increases permeation promoter activity.

Transition from the intra- to inter-molecular process can be controlled by not only molecular structure of the oxidized lipid, but also the flexibility, fluidity, and polarity of other components. This indicates that H_2_O_2_ permeation can be controlled by two independent mechanisms; intra- and inter-molecular processes. From the present study, we have identified two independent permeation routes of H_2_O_2_; (i) permeation through lipid membrane with increased partition coefficient by the intra-molecular configurational change and (ii) diffusion through pores (water channels) formed by the inter-molecular configurational change of the oxidized lipids. We expect that the factors necessary to select a permeable molecule is different between these two mechanisms. The former depends on hydrophilicity of permeating molecules, whereas the latter depends on size of permeating molecules. A rational design of both oxidized lipid and other components based on these findings, will make it possible to control the permeation of not only H_2_O_2_, but also other molecules. We are currently investigating how the molecular structure of the oxidized lipid affects the transition behaviour, the concentration at which the transition occurs, and the permeation ability. This technology opens new avenues for the development of novel methodology in drug delivery^46^ and chemotherapy^47^ that relies on selective permeation of drug molecules.

## Conclusion

We have characterized the permeation behaviour of H_2_O_2_ through DOPC membrane doped with oxidized lipids with different structures. POVPC showed an intra-molecular switching from nonlooped to looped configuration depending on its concentration in the DOPC membrane, which changed the role of POVPC from permeation inhibitor to promoter. The underlying mechanism to promote H_2_O_2_ permeation was explained by the increased partition coefficient of lipid membrane owing to the incorporation of the hydrophilic oxidized group. On the other hand, PazePC underwent an inter-molecular self-assembly, in a similar manner as pore-forming peptides. Pores formed through the self-assembly of PazePC molecules acted as the permeation promoter. In contrast to POVPC, that promoted permeation by increasing partition coefficient of the membrane, enhanced permeation by PazePC was explained by the formation of diffusion passage for H_2_O_2_ through the pore. Contrary to these two oxidized lipids, PoxnoPC acted as a permeation inhibitor under our experimental condition. Thus, we have elucidated two independent mechanisms of permeation of H_2_O_2_; configuration change of oxidized lipids via intra- and inter-molecular processes, which provides new insight into how biological cells control the permeation of molecules through lipid membrane. Furthermore, exploring a biological or rationally designed artificial molecule that undergoes a stimuli-responsive switching between looped, nonlooped, and self-assembled states might make it possible to control release of a drug from liposomes. Switching between looped and nonlooped configuration enables the selective release of drugs depending on their hydrophilicity, whereas the self-assembled pore-formation enables selective release depending on their molecular size.

## Supplementary information

Supplementary Information
