# Supplementary material for: Role of Oxidized Lipids in Permeation of H2O2 Through a Lipid Membrane: Molecular Mechanism of an Inhibitor to Promoter Switch
Source: Sci Rep. 2019 Aug 29;9:12497. doi: 10.1038/s41598-019-48954-z (PMC6715804; doi:10.1038/s41598-019-48954-z)
Supplement: Supplementary file 1 — Supplementary Information [file 41598_2019_48954_MOESM1_ESM.pdf]

Supplementary Information

Role of Oxidized Lipids in Permeation of H_2O_2 Through a Lipid Membrane: Molecular Mechanism of an Inhibitor to Promoter Switch

*Yuya Ouchi, Kei Unoura, Hideki Nabika**

Department of Material and Biological Chemistry, Faculty of Science, Yamagata University, 1-4-12 Kojirakawa, Yamagata 990-8560, Japan

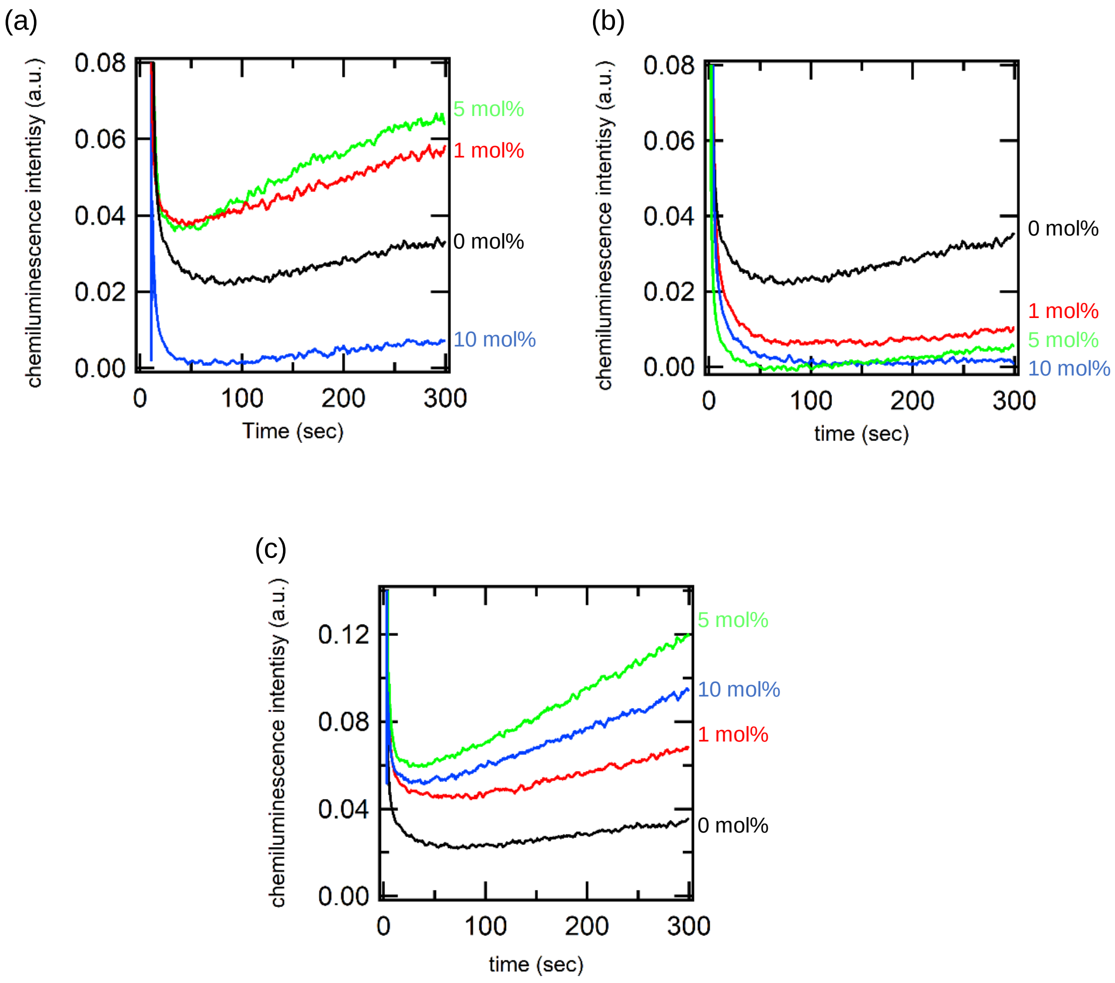

Figure S1. Representative experimental results of luminescence intensity vs time for (a) POVPC, (b) PoxonoPC, and (c) PazePC.

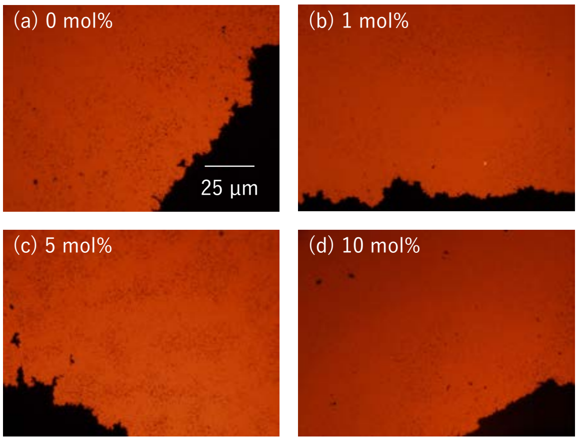

Figure S2. Fluorescence microscopy images of the DOPC membrane doped with POVPC at (a) 0 mol%, (b) 1 mol%, (c) 5 mol%, and (d) 10 mol% after H_2O_2 exposure.
